# Nitrous Oxide Induces Prominent Cell Proliferation in Adult Rat Hippocampal Dentate Gyrus

**DOI:** 10.3389/fncel.2018.00135

**Published:** 2018-05-17

**Authors:** Farah Chamaa, Hisham F. Bahmad, Ahmad-Kareem Makkawi, Reda M. Chalhoub, Elie D. Al-Chaer, George B. Bikhazi, Ziad Nahas, Wassim Abou-Kheir

**Affiliations:** ^1^Department of Anatomy, Cell Biology and Physiological Sciences, Faculty of Medicine, American University of Beirut, Beirut, Lebanon; ^2^Department of Anesthesiology, Faculty of Medicine, American University of Beirut Medical Center, Beirut, Lebanon; ^3^Department of Psychiatry, Faculty of Medicine, American University of Beirut Medical Center, Beirut, Lebanon; ^4^Department of Psychiatry, Medical School, University of Minnesota, Minneapolis, MN, United States

**Keywords:** nitrous oxide, anesthetics, depression, neurogenesis, dentate gyrus, hippocampus

## Abstract

The identification of distinct and more efficacious antidepressant treatments is highly needed. Nitrous oxide (N_2_O) is an N-methyl-D-aspartic acid (NMDA) antagonist that has been reported to exhibit antidepressant effects in treatment-resistant depression (TRD) patients. Yet, no studies have investigated the effects of sub-anesthetic dosages of N_2_O on hippocampal cell proliferation and neurogenesis in adult brain rats. In our study, adult male Sprague-Dawley rats were exposed to single or multiple exposures to mixtures of 70% N_2_O and 30% oxygen (O_2_). Sham groups were exposed to 30% O_2_ and the control groups to atmospheric air. Hippocampal cell proliferation was assessed by bromodeoxyuridine (BrdU) incorporation, and BrdU-positive cells were counted in the dentate gyrus (DG) using confocal microscopy. Results showed that while the rates of hippocampal cell proliferation were comparable between the N_2_O and sham groups at day 1, levels increased by 1.4 folds at day 7 after one session exposure to N_2_O. Multiple N_2_O exposures significantly increased the rate of hippocampal cell proliferation to two folds. Therefore, sub-anesthetic doses of N_2_O, similar to ketamine, increase hippocampal cell proliferation, suggesting that there will ultimately be an increase in neurogenesis. Future studies should investigate added N_2_O exposures and their antidepressant behavioral correlates.

## Introduction

Major depressive disorder (MDD) is considered one of the world’s most disabling illnesses (Collins et al., 2011). According to the World Health Organization (WHO), MDD ranks second in the global burden of disease, accounting for 2.5% of global disability adjusted life years (Ferrari et al., 2013). Despite the usefulness of the currently available antidepressant therapies (UK ECT Review Group, 2003; Mayberg et al., 2005), more than 20% of patients with depression still suffer from a virulent subtype of treatment-resistant depression (TRD; Rush et al., 2006a,b; Mrazek et al., 2014). Novel treatment applications engaging molecular targets are desperately needed to identify faster and more efficacious antidepressant strategies other than the monoamine system.

Accumulating evidences put forward an increased association of N-methyl-D-aspartic acid (NMDA) receptor signaling with the neurobiology of depression (Li et al., 2010; Autry et al., 2011; Duman and Aghajanian, 2012). This subtype of glutamate receptor suggests a novel therapeutic approach for its antidepressant effects (Trullas and Skolnick, 1990; Zarate et al., 2006; Ates-Alagoz and Adejare, 2013; Dang et al., 2014). Accordingly, several trials have reported effective results in using NMDA receptor antagonists such as ketamine, which is commonly used in general anesthesia, (Krystal et al., 1994; Berman et al., 2000; Zarate et al., 2012) for treating depression. However, ketamine can be associated with psychotomimetic side effects including delusions, illusions and hallucinations (Mechri et al., 2001). A pilot trial opted to investigate the therapeutic effects of sub-anesthetic doses of nitrous oxide gas (laughing gas, N_2_O) on TRD patients (Nagele et al., 2015). Results showed N_2_O gas to be well-tolerated, with acute reduction of depressive symptoms that lasted for at least 24 h and up to 1 week in some of the patients (Nagele et al., 2015).

Patients with major depression have a reduced hippocampal volume (Bremner et al., 2000), while stressed animals show decreased neurogenesis in the subgranular zone (SGZ), the germinal layer of the dentate gyrus (DG; Gould et al., 1997). This zone contains granule neurons that arise from neural stem cells and thus keep generating continuously during adulthood (Altman and Das, 1965; Eriksson et al., 1998; Gould et al., 1998). While depression negatively affects neurogenesis in addition to other structural changes in the hippocampus, it is still debatable if impairing neurogenesis would lead to depression (Miller and Hen, 2015). Moreover, modulations of the NMDA system or treatments with various antidepressants have been correlated with increased neurogenesis in the adult hippocampus, mostly in stressed rodents that have increased stress hormone levels (Malberg et al., 2000; Manev et al., 2001; Nacher et al., 2003; Santarelli et al., 2003; David et al., 2009; Miller and Hen, 2015). Although N_2_O gas is known to act as an NMDA receptor antagonist with possible anti-depressant properties (Jevtović-Todorović et al., 1998), its possible influence on hippocampal cell proliferation has not been determined yet. Thus, we leveraged these observations to investigate the possible effects of sub-anesthetic N_2_O doses on hippocampal cell proliferation of stem/progenitor cells in the hippocampal DG in adult brain rats.

## Materials and Methods

### Sprague-Dawley Rats

To examine the hippocampal cell proliferation levels in rats, two separate experiments (one or four sessions gas exposure) were performed on adult male Sprague-Dawley rats (250–300 g) in accordance with the National Institutes of Health Guidelines for Animal Research (Guide for the Care and Use of Laboratory Animals) and under a protocol approved by the Institutional Animal Care and Use Committee (IACUC) at the American University of Beirut (AUB; Zimmermann, 1983). The study with all its experimental protocols was conducted under the Institutional Review Board (IRB) approvals of AUB. All experiments were performed in accordance with relevant guidelines and regulations. Animals were maintained in a controlled environment, temperature (20–22°C), 12 h light/dark cycle and provided with water *ad libitum*. Post-exposure behavioral and body weight monitoring were conducted during the light phase of the cycle by a researcher blind to the treatment conditions.

### Gas Exposure

**Figure 1:**
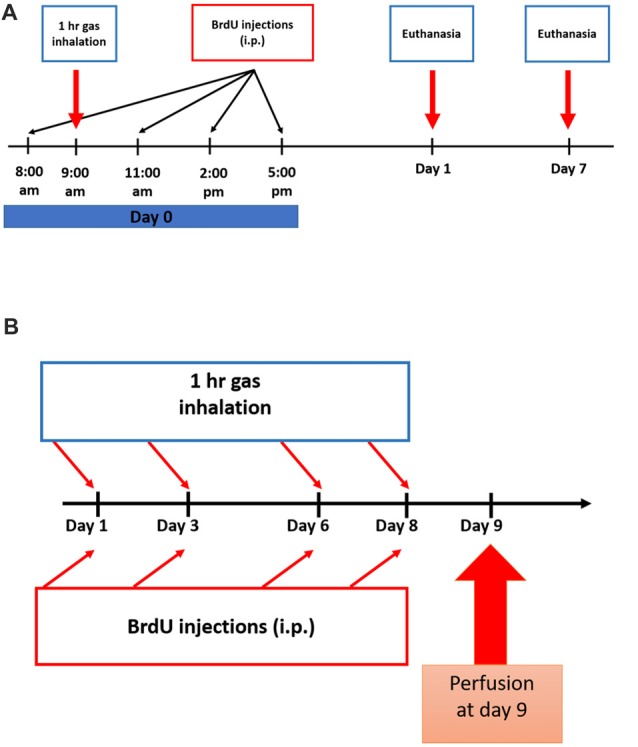
Experimental schedule for Nitrous Oxide (N_2_O) exposures and BrdU injections. **(A)** Scheme of the experimental procedures for single exposure to the gases where rats were sacrificed on days 1 or 7. **(B)** Timeline for multiple exposures to the gases where the animals were sacrificed on day 9.

All rats were placed in a transparent anesthetic chamber at room temperature (RT) for 15 min of acclimatization before gas exposure sessions (1 h duration each). A gas monitor within the chamber measured the gas composition. Subanesthetic dose of N_2_O, in humans as well as in mice and rats, ranges from 10% to 70% (Chambers and Schultz, 1945; Koblin et al., 1979; Yamamura et al., 1981; Frost and Rubin, 2013). In the chamber, the experimental groups were exposed for one or four sessions of 70% N_2_O and 30% O_2_ gas for 1 h (*n* = 4 in the one session groups, *n* = 6 in the four-session group). All rats remained fully awake, not sedated or anesthetized all through the experiments. The sham animals, however, were supplied with 30% O_2_ for the same duration (*n* = 4 in the one session groups, *n* = 6 in the four-session group). A control group was only exposed to atmospheric air in the chamber (*n* = 5 in the four-sessions group). After gas exposure, rats were allowed to completely recover before being returned to their home cages. The timelines of the experiments and inhalation protocol are represented in Figures 1A,B.

### Brdu Administration

To test for the proliferation of stem/progenitor cells, all rats were injected with 5-bromo-2′-deoxyuridine (BrdU, Sigma-Aldrich, 50 mg.kg^−1^, *i.p*.) dissolved in 0.9% warm saline. All groups received a total of four injections as follows: the single-exposure groups received all the injections at day 0 with a 3 h interval between each injection (Figure 1A) and the multiple exposure groups were given one injection per day after each exposure session (Figure 1B). In the single exposure description (Figure 1A), BrdU was given at 8 am, the gas exposure was started at 9 am and continued for 1 h till 10 am. The three subsequent doses of BrdU injections were given afterwards. For multiple exposure (Figure 1B), each BrdU dose (50 mg/kg/ip injection) was given after exposure session by around 15 min.

### Tissue Preparation for Stereology

In order to prepare the tissues for exploration of stem/progenitor cells proliferation, rats were deeply anesthetized by intraperitoneal (ip) injection of ketamine (Ketalar^®^, Panpharma; 50 mg/kg) and xyla (Xylazine^®^, Interchemie; 12 mg/Kg), and perfused transcardially with 0.9% saline and 4% formalin. Brains were removed, fixed overnight in 4% paraformaldehyde and then cryoprotected with 30% sucrose solution for 3 days. Systemic-random sampling of brain sections were completed following the Fractionator principle (Gundersen et al., 1999). In brief, 40 μm coronal sections were cut serially using a freezing microtome, from the rostral to the caudal extent of the DG at the following rostro-caudal coordinates covering the whole hippocampal formation (−2.12 to −6.3 mm relative to bregma). To highlight the topographic correspondence of BrdU distribution, the DG region was divided into three areas as follows: rostral ranging from −2.12 mm to −3.7 mm relative to bregma, intermediate ranging from −3.7 to −4.9 and caudal ranging from −4.9 to −6.3 (Paxinos and Watson, 1998). Sections were serially collected in six sets containing seven rostral, five intermediate and six caudal sections per set. All sections were collected and stored in 0.1 M PBS solution containing sodium azide (15 mM).

### Immunofluorescence

For BrdU detection, DNA was denatured by incubating the sections in 2N HCl for 30 min at 37°C. Sections were rinsed with 0.1 M PBS (Sigma-Aldrich) and washed with 0.1 M Sodium Borate (pH 8.5) for 10 min at RT to neutralize acidic effect. Tissues were washed with 0.1 M PBS and transferred to the blocking and permeabilization solution (10% NGS, 3% BSA, 0.1% Triton-X diluted in PBS) for 1 h at 4°C. In order to minimize non-specific cross labeling between different primary antibodies, we sequentially stained the sections. Therefore, sections were incubated overnight at 4°C with rat monoclonal anti-BrdU (1:100; Bio-Rad) diluted in PBS with 3% BSA and 3% NGS, 0.1% Triton-X. The following day sections were washed and incubated in the dark with fluorochrome-conjugated secondary antibody Alexa Fluor-568 anti-rat (1:200; Invitrogen) diluted in same solution for 2 h at RT on a rotator. Sections were washed and incubated with mouse monoclonal anti-NeuN (1:500; Millipore) at 4°C overnight, for the mature neuronal staining of the hippocampus. Proliferating Cell Nuclear Antigen PCNA (Abcam, 1:100) or Ki 67 (Abcam, 1:500) were used as proliferation markers at Day 1 post nitrous oxide inhalation and the immature neuronal lineage marker Doubelcortin DCX (Abcam, 1:200) was used at Day 7 post inhalation. The next day the secondary antibodies Alexa Fluor-488 anti-mouse and Alexa Fluor-633 anti-rabbit (1:250; Invitrogen) was applied as before and Hoechst stain (Invitrogen) was added for 10 min before the final wash. Finally, sections were mounted onto slides with Fluoro-Gel (Electron Microscopy Sciences, USA) and covered with a thin glass coverslip.

### Imaging and Quantification

Quantitative analysis for the changes in cellular proliferation were assessed using confocal stereology of BrdU labeled cells. One well/set was chosen randomly and BrdU+ cells were counted using 40×-oil objective. Cell stereology were confined to SGZ of the DG. Since the counting was solely done in one representative well/set, the final number of BrdU positive cells in each region (rostral, intermediate or caudal) was multiplied by six to estimate the full count in the specified region (Chamaa et al., 2016). The sum of the final numbers of BrdU+ cells in the rostral, intermediate and caudal regions were added up together to obtain the total number in the two hippocampi of the brain. Microscopic analysis was performed using Zeiss LSM 710 confocal microscope. Cells were counted by a single-blinded researcher and images were acquired and analyzed using the Zeiss ZEN 2012 image-analysis software. Images of BrdU+ cells were acquired under the same laser and microscopic parameters for the purpose of consistency.

### Statistical Analyses

Cell count data were presented as mean ± standard errors. The determination of the significance of differences were done using *t*-test for the single-exposure groups and analysis of variance (ANOVA) for the multiple exposure groups, with significant *p*-value <0.05. ANOVA were followed by Tukey’s multiple comparisons test. Statistical analysis and plotting of figures was done using Prism six GraphPad package (GraphPad software Inc., CA, USA).

## Results

### Single Exposure Session

**Figure 2:**
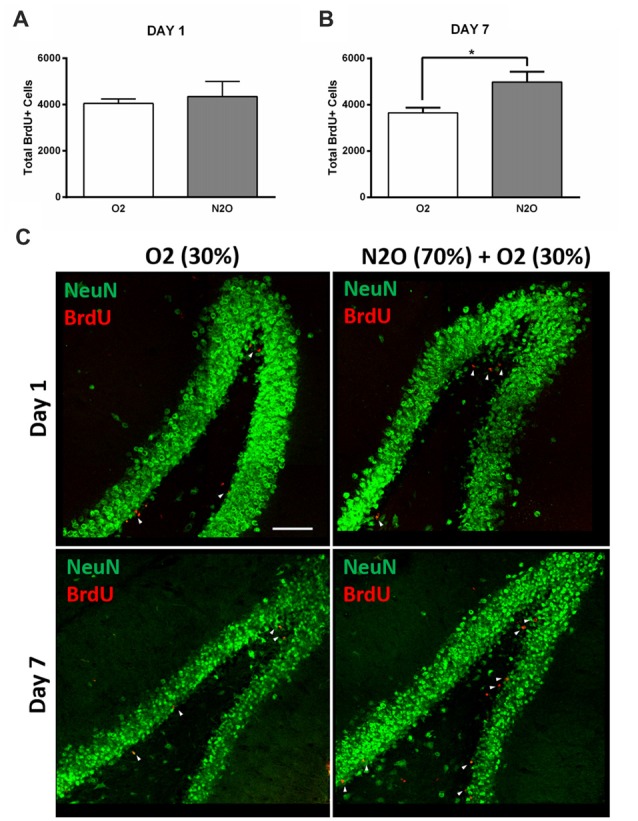
Single exposure to Nitrous Oxide (N_2_O) induces an increase in dentate gyrus (DG) cell proliferation at day 7. **(A,B)** Stereological quantification of BrdU-labeled cells in the DG of adult rats exposed to Oxygen (O_2_) and N_2_O at days 1 and 7 (*n* = 4 each). Each bar represents the average ± SEM of BrdU quantification. The determination of significance of each value was made with reference to the oxygen group using *t*-test (*p < 0.05). **(C)** Representative confocal images showing the DG (green) containing comparable number of BrdU-labeled cells (red) between the two groups at day 1 and higher numbers at day 7 (marked by white arrow heads). Scale bar = 100 μm.

To examine the possible effect on hippocampal cell proliferation, N_2_O mixture (70% N_2_O, 30% O_2_) was tested on preliminary groups for 1 h exposure sessions (*n* = 4 per group). This 1 h of N_2_O mixture inhalation was not sufficient to induce significant changes in stem/progenitor cells proliferation 1 day after the session (Figures 2A,C), however, a significant increase was detected 7 days following exposure, where the rates of BrdU positive cells significantly increased from 3641 ± 233 in 30% O_2_ exposed animals to 4976 ± 451 in N_2_O exposed animals (*p* < 0.05; Figures 2B,C). Most of the BrdU-positive cells at day 1 were immunoreactive with the proliferation markers PCNA and Ki 67 (Supplementary Figure S1A upper and lower panel, respectively). The BrdU positive cells were seen to be co-labeled with the immature neuronal marker DCX at day 7 (Supplementary Figure S1B). No signal was detected when the sections were probed with secondary antibodies alone (Supplementary Figure S2).

### Multiple Exposure Sessions

**Figure 3:**
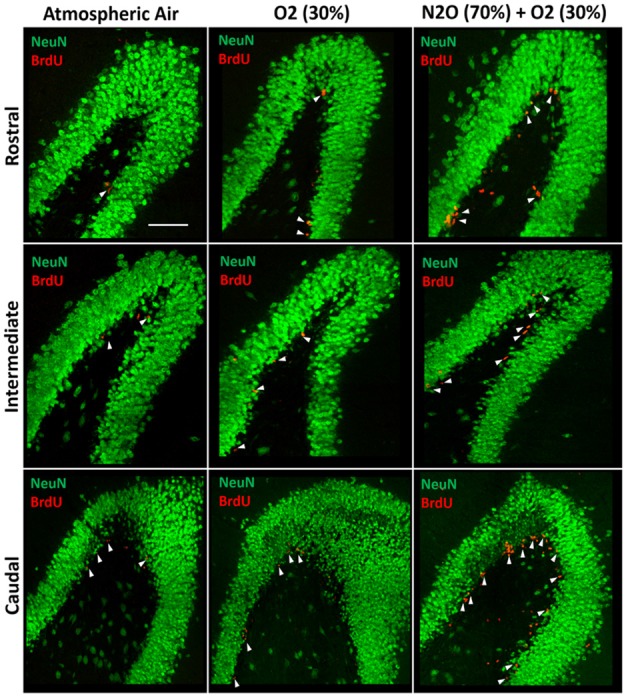
Spatial distribution of the total number of BrdU-labeled cells in the DG following multiple exposures of either atmospheric air, O_2_ 30% or nitrous oxide (70% N_2_O/30% O_2_). Immunofluorescence labeling of rostral, intermediate and caudal DG by NeuN (green) and BrdU (red) showing the spatial distribution of the BrdU-positive cells in the different groups. Scale bar = 50 μm.

**Figure 4:**
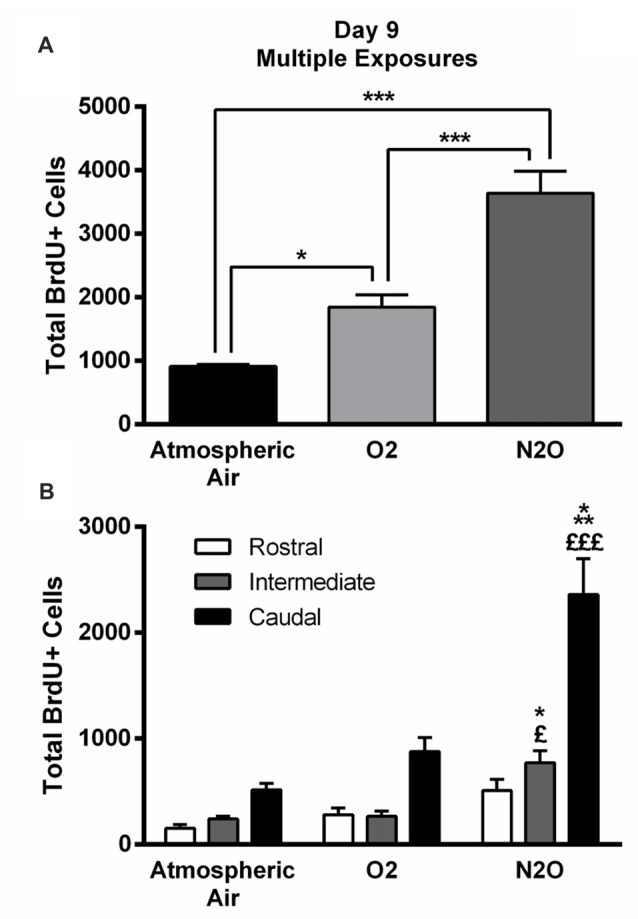
Four separate 1-h exposures to Nitrous Oxide (N_2_O) induces increased neurogenesis in the DG of the hippocampus at day 9. **(A)** The total number of BrdU-positive cells in the hippocampus significantly increased following multiple exposures to N_2_O and was partially increased following exposure to O_2_ (30%). **(B)** Spatial distribution of the total number of BrdU-labeled cells in rostral, intermediate and caudal segments of the DG in control (atmospheric air) (*n* = 5), sham (O_2_ 30%) (*n* = 6) and experimental (70% N_2_O/30% O_2_) rats (*n* = 6). The most prominent increase in the N_2_O group was in the intermediate and caudal regions of the hippocampus. The determination of significance of each value was made with reference to the atmospheric air group (*) or the oxygen group (£). Each bar represents the average ± SEM of BrdU quantification. The determination of significance of each value was made using ANOVA followed by Tukey’s *post hoc* test (*p < 0.05, ***p < 0.001, ^£^*p* < 0.05,^£££^*p* < 0.001).

The significant increase in proliferation following one N_2_O mixture exposure may predict even greater effects following multiple exposure examination. N_2_O mixture was inhaled for 1 h per day at a period of 4 days and the proliferation in DG was examined at day 9. Results showed significant surge in BrdU positive cells (3640 ± 346) in N_2_O mixture exposed rats as compared to 1842 ± 199 in the O_2_ alone exposed animals (*p* < 0.001) and 910 ± 35 in the rats exposed to atmospheric air (*p* < 0.001; Figures 3, 4A). N_2_O mixture increased the proliferation rate of stem/progenitor cells to two folds of the O_2_ exposed group (sham) and to four folds of the atmospheric air exposed group (control).

Of note, there was an increase in BrdU positive cells counted following exposure to 30% O_2_. The number was 910 ± 35 in rats exposed to atmospheric air, but multiple sessions of 30% O_2_ exposure significantly increased the count to 1842 ± 199 (*p* = 0.04; Figures 3, 4A) inducing a 2-fold increase.

### Spatial Distribution of Stem/Progenitor Cells

The BrdU positive cells were counted in each region of the hippocampus; rostral, intermediate and caudal. The highest numbers of stem/progenitor cells were typically found in the caudal regions and the most notable exposure effects were confined to it and to the intermediate regions (Figures 3, 4B). Multiple exposures to sub-anesthetic doses of N_2_O mixture increased the number of BrdU positive cells in the caudal hippocampus to 2361 ± 337 while it was 516 ± 63 in rats exposed to atmospheric air (*p* < 0.001) and 878 ± 134 in rats exposed to O_2_ (*p* < 0.001). The increase was also significant in the intermediate region (770 ± 117 in N_2_O group vs. 242 ± 25 in atmospheric air group, *p* < 0.05, and 265 ± 52 in O_2_ group, *p* < 0.05; Figures 3, 4B).

## Discussion

In this study, we have demonstrated that N_2_O exposure at sub-anesthetic doses can significantly increase hippocampal cell proliferation in the short term, suggesting increased neurogenesis in adult rats. One session of N_2_O significantly increased the proliferation of stem/progenitor cells in the DG, and an accumulation of exposure sessions, putatively mimicking future applications in clinical settings, collectively induced a surge in hippocampal cell proliferation. Evidence from studies dating back in the late 1990s reveal, in agreement with our results, that NMDA receptor blocking by different antagonists such as MK-801 (Gould et al., 1997) and CGP-43487 (Nacher et al., 2003) increase hippocampal neurogenesis. Ketamine, however, had varying effects whereby sub-anesthetic doses (Keilhoff et al., 2004) increased DG neurogenesis while higher anesthetic doses caused inhibition of hippocampal neurogenesis in young rats (Huang et al., 2016).

Our data showed a significant increase in hippocampal cell proliferation when rats were exposed to a concentration of oxygen (30%) that is slightly higher than that of atmospheric air. Previous studies in the literature have reported that exposure to 100% oxygen (hyperbaric oxygen therapy, HBOT) induces neurogenesis in different models (Yang et al., 2008; Wang et al., 2009; Zhang et al., 2011; Liu et al., 2016), but none have reported any information regarding lower oxygen concentrations and increased cell proliferation.

When screening the DG as three rostro-intermediate-caudal regions, we observed a spatial distribution of cell proliferation dispersed as follows: the rostral region contained the fewest number of proliferating stem/progenitor cells, the numbers were elevated in the intermediate region and were highest in the caudal region. This spatial distribution was detected in all groups of animals and is in line with previous data from our laboratory (Chamaa et al., 2016). With exposure to multiple sessions of 70% N_2_O/30% O_2_, this spatial pattern was conserved and increased over all regions but most significantly in the intermediate and the caudal area. The most common terms for hippocampal divisions used in the literature are the dorsal and ventral hippocampus. Several studies have correlated changes in neurogenesis to the ventral hippocampus in animal models of depression (Brummelte and Galea, 2010; Elizalde et al., 2010; Oomen et al., 2010; Morley-Fletcher et al., 2011; Tanti et al., 2012). In our study, both the intermediate and the caudal regions can be considered as part of the ventral hippocampus reiterating the importance of N_2_O gas in affecting the same areas of stem/progenitor cell proliferation as depression. Further investigation of the spatial distribution of cell proliferation in depression models and in its potential treatments is highly important for the functional implications of depression-neurogenesis-anatomical preferences.

Nagele’s “proof-of-concept” trial (Nagele et al., 2015) explored N_2_O as a rapid antidepressant intervention given the intimate link between NMDA receptor signaling and neurobiology of depression (Berman et al., 2000; Li et al., 2010; Autry et al., 2011; Duman and Aghajanian, 2012). Indeed, ketamine and other NMDA antagonists have been studied in several trials to assess their efficacy in treating depression (Krystal et al., 1994; Berman et al., 2000; Zarate et al., 2006; Phelps et al., 2009; Zarate et al., 2012). While both ketamine and N_2_O gas have been shown to augment excitatory synaptic function in certain brain regions like the hippocampus and frontal cortex (Zorumski et al., 2015), ketamine presented with significant psychotomimetic side effects (Mechri et al., 2001) whereas N_2_O gas was generally well-tolerated (Zorumski et al., 2015). At the cellular level, effects of N_2_O gas, and unlike ketamine, are also found to be less voltage-dependent with no decline in the NMDA receptor-mediated synaptic currents (Mennerick et al., 1998) as N_2_O is unlikely to have large presynaptic effects on glutamate transmission (Zorumski et al., 2015). However, at higher anesthetic concentrations and like other NMDA antagonists, N_2_O possesses neurotoxic side effects mainly caused by inhibition of ionic currents. This can be prevented by drugs enhancing GABAergic inhibition (Jevtović-Todorović et al., 1998). Collectively, this presents the importance of N_2_O to be extensively studied in depression models, specifically TRMD.

Albeit, it is still unclear whether neurogenesis should be a primary target in treating depression (Henn and Vollmayr, 2004). On one hand, recent studies have highlighted an association between new DG neurons and antidepressant treatments (Santarelli et al., 2003; Airan et al., 2007; Surget et al., 2008, 2011). Further studies have correlated depressive-like behavior with decreased neurogenesis under stressful conditions (Lehmann et al., 2013) in addition to associating attenuation of depressive-like behavior with increased neurogenesis (Malberg et al., 2000; Manev et al., 2001; Nacher et al., 2003; Santarelli et al., 2003; Encinas et al., 2011; Hill et al., 2015), particularly in the SGZ (Gould et al., 1997). On the other hand, other studies have suggested no correlation between effective antidepressant treatments and neurogenesis (Vollmayr et al., 2003; Henn and Vollmayr, 2004). Therefore, whether the process of neurogenesis is necessary for mediating the effectiveness of antidepressants or not still needs further investigation.

In summary, this study investigated the effect of exposure to N_2_O gas on cell proliferation, considered as first stages of hippocampal neurogenesis, in non-stressed animals and found that it enhances proliferation of stem/progenitor cells. The findings are in line with the neurogenic effects of other NMDA receptor antagonists and add to the increased interest in utilizing N_2_O gas as a noninvasive antidepressant treatment modality. Future studies are needed to specifically follow up on the survival and fate of these proliferating cells whereby a quantification of BrdU-DCX+ cells at early time points and that of BrdU-NeuN+ cells at later time point is a must. This will clearly show the fate of the newly proliferating cells as they integrate into the granular cell layer of the DG in the hippocampus. Complete studies should be executed to assess the effect of N_2_O gas inhalation on behavior in animal models of depression and anxiety.

## Author Contributions

FC, HB, A-KM, RC and WA-K contributed to the project design and execution of experiments, analysis of results and writing of manuscript. GB, ZN, EA-C and WA-K contributed to overlooking and following up with experiments, result analysis and manuscript proofreading. WA-K contributed to project design, result analysis, manuscript writing and proofreading. GB, ZN, EA-C and WA-K critically revised and edited the manuscript. All authors have read and approved the final draft.

## Conflict of Interest Statement

The authors declare that the research was conducted in the absence of any commercial or financial relationships that could be construed as a potential conflict of interest.

## Supplementary Material

FIGURE S1 Co-localization of BrdU-labeled cells with proliferation markers at day 1 and neuronal lineage marker at day 7 following single exposure. **(A)** Confocal images of BrdU-labeled cells co-localized with PCNA (upper panel) and Ki 67 (lower panel) at day 1. **(B)** Confocal images of BrdU-labeled cells co-localized with DCX at day 7. Co-localization marked by white arrow heads. Scale bars: 50 μm in enlarged images and 20 μm in the insets.

FIGURE S2 Immuno-reactivity of the secondary antibodies in the DG. **(A)** Representative confocal image showing no immuno-reactivity of the secondary antibody Alexa-563 conjugated Anti-rat Igg. **(B)** Representative confocal image showing no immuno-reactivity of the secondary antibody Alexa-488 conjugated Anti-mouse Igg. **(C)** Representative confocal image showing no immuno-reactivity of the secondary antibody Alexa-633 conjugated Anti-rabbit Igg. Scale bars: 100 μm.
