# Supplementary figures and images for: Nitrous Oxide Induces Prominent Cell Proliferation in Adult Rat Hippocampal Dentate Gyrus
Source: Front Cell Neurosci. 2018 May 17;12:135. doi: 10.3389/fncel.2018.00135 (PMC5967150; doi:10.3389/fncel.2018.00135)

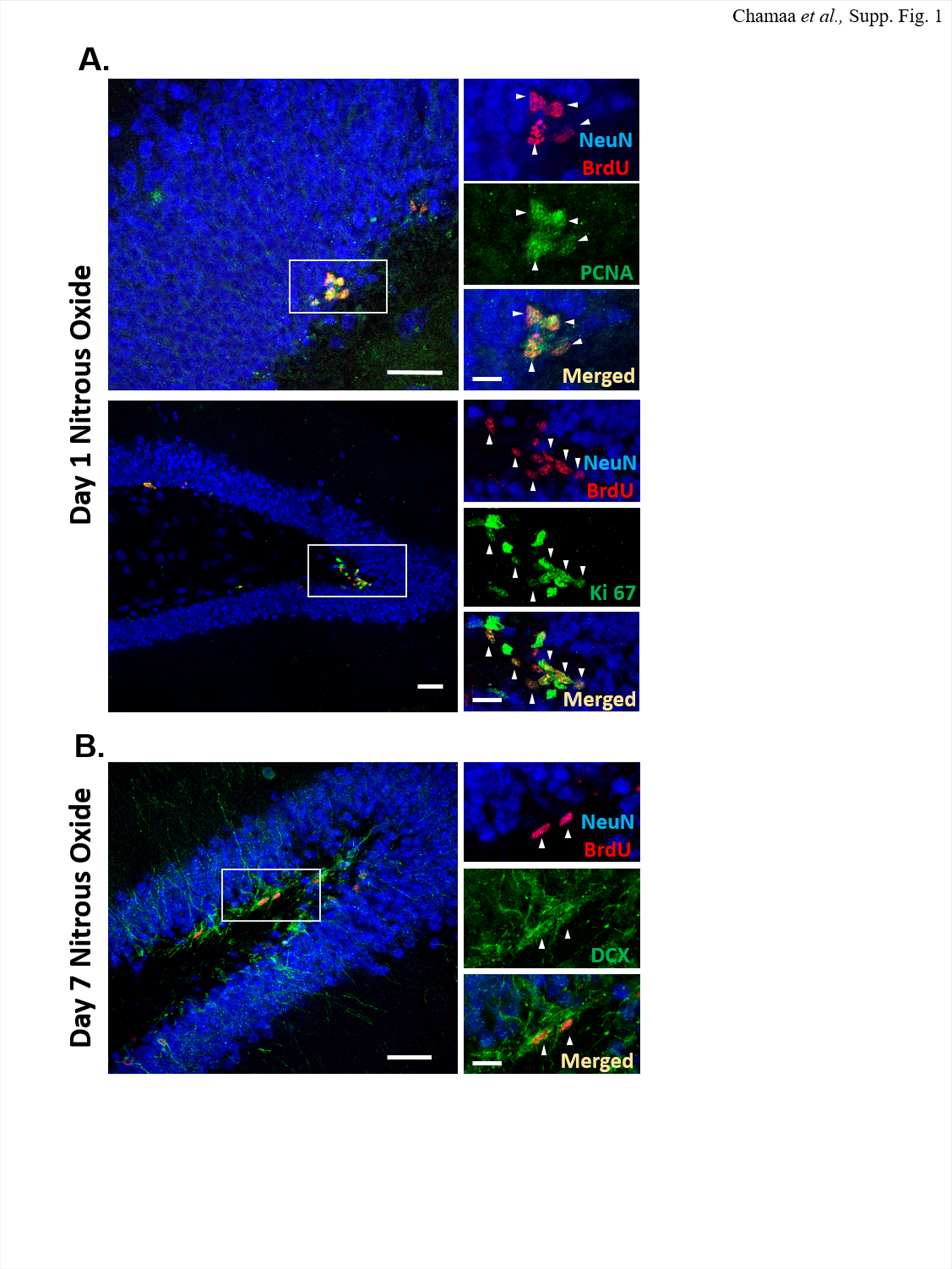

Supplement: FIGURE S1 — Co-localization of BrdU-labeled cells with proliferation markers at day 1 and neuronal lineage marker at day 7 following single exposure. (A) Confocal images of BrdU-labeled cells co-localized with PCNA (upper panel) and Ki 67 (lower panel) at day 1. (B) Confocal images of BrdU-labeled cells co-localized with DCX at day 7. Co-localization marked by white arrow heads. Scale bars: 50 μm in enlarged images and 20 μm in the insets. [file Image_1.TIF]

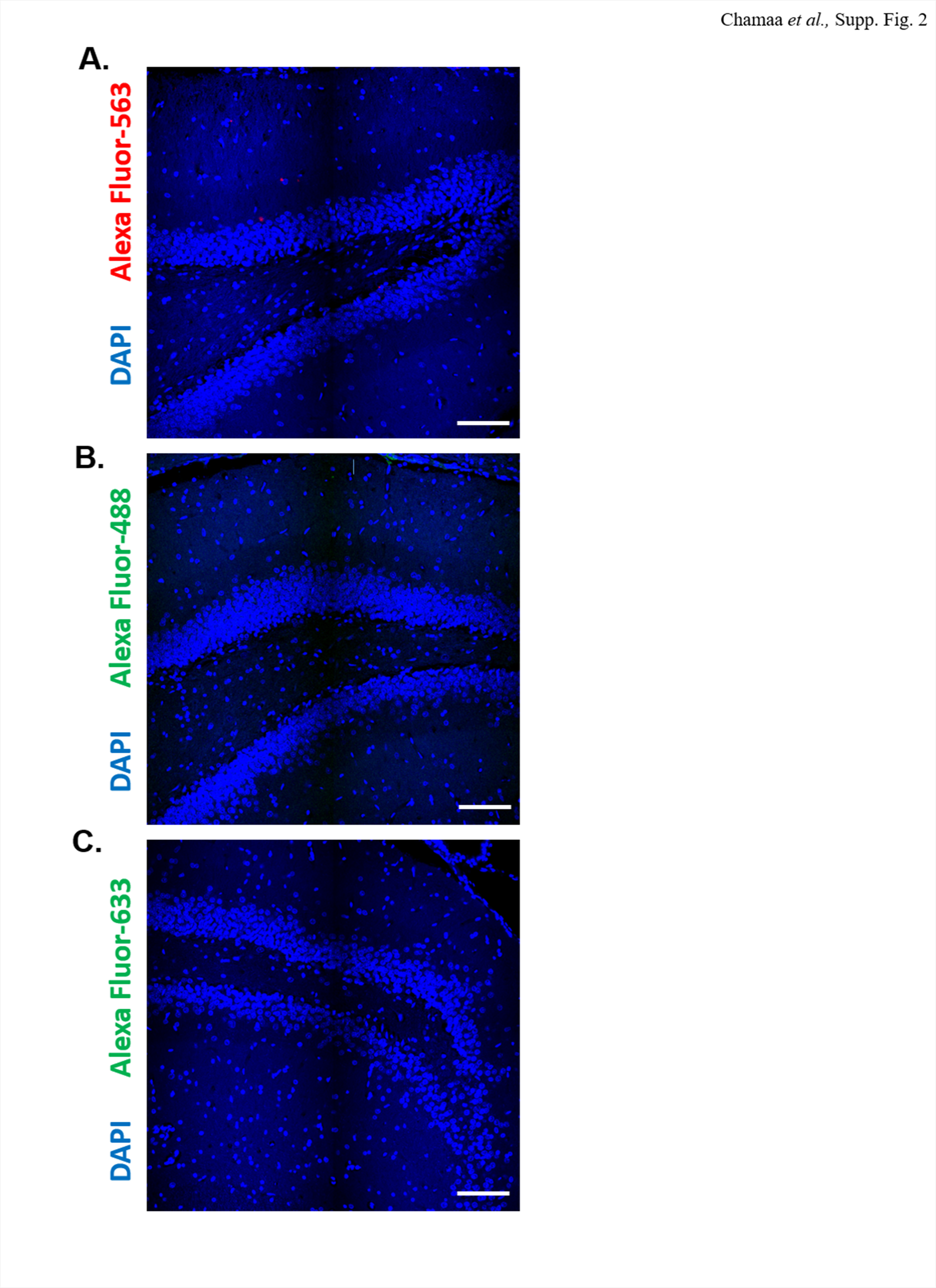

Supplement: FIGURE S2 — Immuno-reactivity of the secondary antibodies in the DG. (A) Representative confocal image showing no immuno-reactivity of the secondary antibody Alexa-563 conjugated Anti-rat Igg. (B) Representative confocal image showing no immuno-reactivity of the secondary antibody Alexa-488 conjugated Anti-mouse Igg. (C) Representative confocal image showing no immuno-reactivity of the secondary antibody Alexa-633 conjugated Anti-rabbit Igg. Scale bars: 100 μm. [file Image_2.TIFF]
